# Incidence and Clinical Associations With Phenotypic Drift From Pulmonary Arterial Hypertension to Combined Precapillary and Postcapillary Pulmonary Hypertension

**DOI:** 10.1016/j.chpulm.2025.100206

**Published:** 2025-08-19

**Authors:** Kevin T. Schwalbach, Jeffrey Annis, Jonah David Garry, Hui Nian, Evan L. Brittain, Anna R. Hemnes

**Affiliations:** a Divisions of Allergy, Pulmonary and Critical Care Medicine, Vanderbilt University Medical Center, Nashville, TN; b Divisions of Cardiovascular Medicine, Vanderbilt University Medical Center, Nashville, TN; c Vanderbilt Institute for Clinical and Translational Research, Vanderbilt University Medical Center, Nashville, TN; d Department of Biostatistics, Vanderbilt University Medical Center, Nashville, TN

**Keywords:** phenotypic drift, pulmonary arterial hypertension, pulmonary capillary wedge pressure, pulmonary hypertension

## Abstract

**Background:**

Pulmonary hypertension (PH) diagnosis relies on hemodynamic measurements from right heart catheterization (RHC). Pulmonary arterial hypertension (PAH) (group 1) is differentiated from group 2 PH (due to left-sided heart disease) by pulmonary capillary wedge pressure (PCWP), with group 2 requiring PCWP > 15 mm Hg. Contemporary PAH cohorts are older with more cardiovascular comorbidities compared with early descriptions, making it harder to distinguish between PAH and group 2 PH. It is unclear whether hemodynamic shifts between PH classifications occur over time or how these shifts impact outcomes.

**Research Question:**

How frequently do patients with PAH develop combined precapillary and postcapillary PH (CpcPH), marked by elevated PCWP on follow-up RHC? What baseline characteristics are associated with this change, and does it affect outcomes?

**Study Design and Methods:**

This retrospective cohort analysis at a single academic institution examined demographic, clinical, and RHC data from diagnostic and most recent RHCs. The primary outcome was the incidence of phenotypic drift from PAH to CpcPH. Secondary objectives included identifying clinical features associated with phenotypic drift and its impact on all-cause mortality and hospitalizations.

**Results:**

Of 257 patients with PAH, 58 (22.6%; 95% CI, 17.6%-28.2%) experienced phenotypic drift, whereas 199 (77.4%; 95% CI, 72%-83%) retained PAH hemodynamics. Those with drift were more likely to be Black (29.3% vs 14.1%), have atrial fibrillation (12.1% vs 3.5%), have diabetes (22.4% vs 10.5%), and have higher BMI (30.2 vs 28.1 kg/m^2^). Baseline hemodynamics and echocardiographic features were similar between cohorts. There was no significant difference in survival or time to hospitalization between drift and nondrift groups.

**Interpretation:**

Our results indicate that phenotypic drift from PAH to CpcPH is common and associated with features of metabolic syndrome and atrial fibrillation, but not predicted by baseline RHC or imaging features. Phenotypic drift was now shown to affect survival or hospitalization outcomes and should not routinely influence initial PAH treatment decisions.

**Take-Home Points**

**Study Question:** How frequently do patients with pulmonary arterial hypertension (PAH) develop combined precapillary and postcapillary pulmonary hypertension, what baseline characteristics are associated with this change, and does it affect clinical outcomes?

**Results:** Approximately one-fourth of individuals with PAH develop combined precapillary and postcapillary pulmonary hypertension over time, a phenomenon associated with atrial fibrillation and features of metabolic syndrome, but no differences in baseline echocardiographic features, hemodynamics, or differences in time to first hospitalization or survival compared with those who do not undergo this phenotypic drift.

**Interpretations:** Our results suggest that phenotypic drift defined by newly elevated pulmonary capillary wedge pressure is common in patients with initial diagnosis of PAH but does not affect certain clinically important outcomes and should not routinely inform initial treatment decisions in PAH.

Pulmonary hypertension (PH) is a complex cardiopulmonary disease with multiple etiologies defined by an elevation in mean pulmonary arterial pressures (mPAP) to > 20 mm Hg. In practice, PH is classified into 5 distinct clinical groups based on similar pathophysiological mechanisms and therapeutic management.^1^ Distinguishing between pulmonary arterial hypertension (PAH) (group 1 PH) and group 2 PH related to left-sided heart disease requires invasive hemodynamics.^1^ Although both share an mPAP > 20 mm Hg, PAH requires a pulmonary capillary wedge pressure (PCWP) ≤ 15 mm Hg and elevation in pulmonary vascular resistance (PVR) of ≥ 3 Wood units (WU). Group 2 PH requires a PCWP > 15 mm Hg. It is presently unknown if ever and how commonly there is shift over time from PAH to group 2 PH.

Cardiovascular (CV) comorbidities were rare in early descriptions of PAH cohorts.^2^ More recent reports suggest changes in PAH epidemiology such that patients are now older at diagnosis and have a higher burden of cardiometabolic risk factors.^3^^,^^4^ This trend mirrors rising rates of obesity, diabetes, and CV disease in the general population.^5^ Moreover, evidence from Pulmonary Vascular Disease Phenomics (PVDOMICs) has suggested that nearly 40% of individuals with mostly prevalent PH have > 1 classification, so-called mixed etiology PH.^6^^,^^7^ Specifically, upward of 35% of individuals with PAH may also have features of group 2 PH in this cross-sectional study, possibly leading to attenuation of treatment response and different clinical outcomes.^7^, ^8^, ^9^, ^10^, ^11^ Clinicians have anecdotally observed phenotypic drift from PAH to combined precapillary and postcapillary pulmonary hypertension (CpcPH) over time, but the incidence of this hemodynamic evolution and clinical predictors have not been reported or well defined.

We hypothesized that a subset of patients with PAH experience phenotypic drift into CpcPH over time defined by a new elevation in their PCWP. To test this, we conducted a retrospective analysis of a longitudinal, single-center cohort of patients with PAH. We aimed to identify clinical features at the time of PAH diagnosis associated with phenotypic drift and assess its impact on the clinically important outcomes of all-cause mortality and rates of hospitalization.

## Study Design and Methods

We performed a retrospective cohort analysis at a single academic institution between the years 1995 and 2020. All data were collected from Vanderbilt University’s deidentified electronic health record, the Synthetic Derivative.^12^^,^^13^ The Vanderbilt institutional review board approved this study under a waiver of consent because all data are deidentified (IRB 212147).

### Inclusion and Definition

We used a previously described machine learning PAH algorithm to identify patients with possible PAH^14^ (e-Appendix 1). From this cohort, we then identified patients > 18 years of age, with both diagnostic and follow-up right heart catheterization (RHC) separated by at least 3 months. In patients with > 1 follow-up RHC, we selected the latest available. Hemodynamic criteria for inclusion were mPAP > 20 mm Hg, PCWP ≤ 15 mm Hg, and PVR ≥ 3 WU at diagnostic RHC. This PVR definition corresponds to guidelines before updates in 2022, which changed to PVR ≥ 2 WU, reflecting our period of primary analysis and definitions used in modern clinical trials.^1^^,^^15^ Each case was then manually reviewed to ensure they met this standard PAH hemodynamic definition. Patients not meeting these criteria or with incomplete RHC data as defined by absence of mPAP, PCWP, or PVR after manual review were excluded from analysis. At our institution, all patients with precapillary PH undergo provocative testing with a fluid challenge after baseline hemodynamics are recorded as previously described^16^^,^^17^ (e-Appendix 1).

Demographics, comorbidity data, and laboratory values closest to the date of diagnostic RHC were included. Race and other demographic data were identified by administrative clinical records. We used previously validated algorithms to identify comorbid and laboratory data^14^ (e-Appendix 1).

Methods of extrapolating echocardiographic data from the Synthetic Derivative have been described previously.^18^ Echocardiograms were interpreted by board-certified cardiologists and performed for clinical use; images were not available for review. A broad list of echocardiographic parameters were available. We chose for analysis those parameters frequently used in clinical practice to determine severity of PH and identify features associated with group 2 PH: left ventricular ejection fraction, right ventricular (RV) systolic pressure, left atrial diameter, qualitative RV functional assessment, and diastolic function (e-Appendix 1).

**Figure 1:**
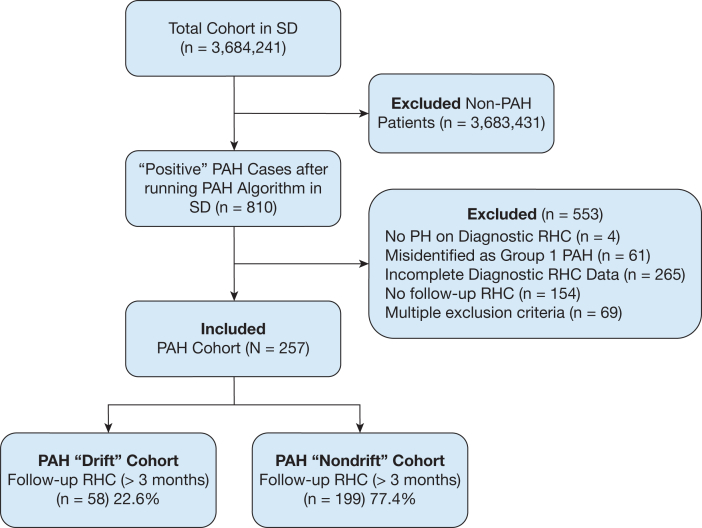
Flowchart of patient inclusion and exclusion. Incidence of phenotypic drift in the study population. SD = Synthetic Derivative; PAH = pulmonary arterial hypertension; PH = pulmonary hypertension; RHC = right heart catheterization.

Patients with PAH with follow-up RHC who demonstrated a resting baseline PCWP > 15 mm Hg were defined as having phenotypic drift (Fig 1). The 2019 World Symposium on Pulmonary Hypertension differentiated group 2 PH into CpcPH and isolated postcapillary PH based on the presence of elevation in PVR ≥ 3 WU in CpcPH in addition to elevation in pulmonary artery pressure and wedge pressure.^15^ We used this standard definition in our analysis.

### Outcomes

Our primary outcome of interest was the incidence of phenotypic drift after a diagnosis of PAH. Secondarily, we compared hemodynamic, clinical, and echocardiographic data between cohorts to determine baseline features associated with phenotypic drift.

We also compared survival and time to first hospitalization between individuals with and without phenotypic drift. Survival was defined as time from diagnostic RHC to time of death from any cause. The Synthetic Derivative algorithm queries death certificates nationally through the date December 31, 2017. After this date, the Synthetic Derivative algorithm is only able to identify those patients who died at Vanderbilt Hospitals through December 31, 2022. To increase accuracy of death status, a manual chart review accompanied the Synthetic Derivative algorithm query to determine whether any clinical documentation was provided to indicate a death occurred outside of the Vanderbilt Health System. Any patient alive or lost to follow-up at time of last electronic medical record query on December 31, 2022, was censored.

Time to first hospitalization was defined as time from initiation of any US Food and Drug Administration-approved PAH specific therapy to first hospital admission. We did not include calcium channel blockers in this category. Because comorbidities may develop differently in drift and nondrift populations over time, we report both time to hospitalization for any cause and also hospitalizations specifically for clinical decompensation due to PH separately. The Synthetic Derivative is only able to query for admissions to our institution. A manual chart review was performed of admission documentation in addition to discharge diagnosis to more acutely determine the reason for hospitalizations. We excluded from analysis any admission to the hospital < 6 weeks from initiation of PAH therapy to avoid applying a definition of clinical worsening to patients who had admissions solely for the reason of initiation or titration of first PAH therapy. These patients were still included in analysis if they had a subsequent admission > 6 weeks from initiation of PAH therapy. Admissions for initiation and titration of parenteral prostacyclins were included if this occurred as add-on therapy for patients previously stable on outpatient oral therapy only. In this cohort, no deaths occurred before first hospitalization.

### Statistical Analysis

Descriptive statistics were presented as mean ± SD or median (interquartile range [IQR]) for continuous variables and frequency (proportion) for categorical variables. Between-group comparison was made using Wilcoxon rank sum test for continuous variables and Pearson χ^2^ test for categorical variables. Missing data for descriptive statistics were handled using listwise deletion. The log-rank test was performed to compare overall survival and time to hospitalization between the groups. The Cox proportional hazard model was then used to investigate the association between phenotypic drift with overall survival and hospitalization with adjustment for age, ethnicity, sex, and baseline PCWP. Missing data for Cox regression analysis were handled via multiple imputation. Analyses were conducted using software PRISM (GraphPad Software) and R 4.3.1 (R: A Language and Environment for Statistical Computing).

## Results

Our algorithm identified a total of 810 individuals that met criteria for PAH at diagnosis from a total cohort of 3,684,241 with records in Vanderbilt’s medical system (Fig 1). A total of 265 patients were excluded because of incomplete RHC data and 154 because of absence of follow-up RHC at Vanderbilt. After manual review, 65 patients were identified as having PAH but did not meet our strict hemodynamic definitions. A total of 69 patients had multiple exclusion criteria. This left a total of 257 patients included in this cohort. Of this cohort, 58 patients (22.6%; 95% CI, 17.6%-28.2%) experienced a phenotypic drift as previously defined through elevation of their PCWP to > 15 mm Hg at follow-up RHC. The remaining 199 patients (77.4%; 95% CI, 72%-83%) retained the hemodynamics of PAH as defined by mPAP > 20 mm Hg and PVR ≥ 3 WU without elevation in PCWP on follow-up RHC.

Characteristics of the drift and nondrift cohorts at diagnosis are shown in Table 1. At diagnosis, the drift cohort had a significantly higher proportion of Black patients (29.3% vs 14.1%; *P* < .01). The drift cohort was also more likely to have atrial fibrillation (AF) (12.1% vs 3.5%; *P* = .01), a higher BMI (30.2 [IQR, 26.4-37.5] vs 28.1 [IQR, 24.0-32.3] kg/m^2^; *P* < .01), and a higher random glucose value (97.5 [IQR, 82-113] vs 91 [IQR, 82-107] mg/dL; *P* = .01). Although the absolute values of hemoglobin A1c did not significantly differ between drift and nondrift cohorts (5.9% [IQR, 5.6%-6.6%] vs 5.6% [IQR, 5.3%-6.1%]; *P* = .05), the proportion of patients with prediabetes (32.8% vs 16.6%; *P* < .01) and diabetes (22.4% vs 10.5%; *P* = .02) was significantly higher in the drift cohort.

**Table 1:** Baseline Characteristics at Study Inclusion

**Variable**	All PAH	Drift	Nondrift	*P* Value
	(N = 257)	(n = 58)	(n = 199)	
**Age**, y	50.6 (40.9-59.1)	48.3 (40.9-58.2)	51.2 (41.1-59.5)	.58
**Female sex**	198 (77.0)	46 (79.3)	152 (76.4)	.64
**Race**				
White	204 (79.4)	40 (69.0)	164 (82.4)	.03
Black	45 (17.5)	17 (29.3)	28 (14.1)	< .01
Other	8 (3.1)	1 (1.7)	7 (3.5)	.17
**Comorbidities**				
AF	14 (5.4)	7 (12.1)	7 (3.5)	.01
Any CTD	97 (37.7)	18 (31.0)	79 (39.7)	.19
Scleroderma	46 (17.9)	6 (10.3)	40 (20.1)	.08
Lupus	50 (19.5)	11 (19.0)	39 (19.6)	.86
MCTD	1 (0.4)	1 (1.7)	0 (0)	NA
OSA	25 (9.7)	4 (6.9)	21 (10.6)	.12
Stroke	2 (0.8)	1 (1.7)	1 (0.5)	NA
Prediabetes	52 (33.5)	19 (32.8)	33 (16.6)	< .01
Diabetes	34 (21.9)	13 (22.4)	21 (10.5)	.02
CAD	37 (14.4)	11 (19.3)	26 (13.1)	.26
HTN	69 (26.8)	20 (34.5)	49 (24.6)	.14
ESRD	14 (5.5)	3 (5.2)	11 (5.5)	.89
Cirrhosis	24 (9.3)	4 (6.9)	20 (10.1)	.47
**Laboratory data**				
BMI (kg/m2)	28.5 (24.5-33.4])	30.2 (26.4-37.5)	28.1 (24.0-32.3)	< .01
BNP (pg/mL)	203 (62.5-542.5)	188 (79.5-365.5)	211 (60-548)	.78
Cr (mg/dL)	0.96 (0.83-1.1)	1.0 (0.84-1.2)	0.94 (0.83-1.1)	.64
eGFR (mL/min/1.73 m^2)^	72.0 (60-87.2)	72.0 (58.1-87.7)	72.0 (60-87.1)	.72
Glucose (mg/dL)	92 (82-107)	97.5 (81.5-113)	91 (82-106.5)	.01
HbA1c (%)	5.8 (5.3-6.2)	5.9 (5.6-6.6)	5.6 (5.3-6.1)	.05
HDL (mgdL)	42 (31-52)	36 (29-48)	44 (32-52)	.14
LDL (mg/dL)	93 (68.5-116.5)	89 (66-99)	97 (72.5-121.5)	.25
Tgs (mg/dL)	109 (84-160)	121.5 (86-181)	107 (84-157)	.97
**TTE**				
LVEF, %	55.5 [6.7]	55.5 [5.3]	55.4 [7.1]	.95
LA diameter, cm	3.6 [0.7]	3.7 [0.7]	3.6 [0.7]	.29
RVSP, mm Hg	79.3 [23.8]	79.1 [25.5]	79.3 [23.3]	.94
**RV dysfunction**				
Any	171 (72.2)	43 (78.2)	128 (70.3)	.25
Mild/moderate	125 (48.6)	32 (55.2)	93 (46.7)	.36
Severe	46 (17.9)	11 (19.0)	35 (17.6)	.90
Diastolic dysfunction	41 (15.9)	9 (15.5)	32 (16.0)	.93

Data are presented as mean [SD], median (interquartile range), No. (%), or as otherwise indicated. The category "other" includes any race that is not defined as "White" or "Black"; AF = atrial fibrillation; BNP = B-type natriuretic peptide; CAD = coronary artery disease; Cr = creatinine; CTD = connective tissue disease; eGFR = estimated glomerular filtration rate; ESRD = end stage renal disease; HbA1c = glycated hemoglobin; HDL = high-density lipoprotein; HTN = hypertension; LA = left atrial; LDL = low-density lipoprotein; LVEF = left ventricular ejection fraction; MCTD = mixed connective tissue disease; PAH = pulmonary arterial hypertension; RV = right ventricular; RVSP = right ventricular systolic pressure; Tgs = triglycerides; TTE = transthoracic echocardiogram.

Comparison of echocardiographic variables at diagnosis demonstrated that the drift and nondrift cohorts had no significant differences in commonly used metrics to distinguish PAH from group 2 PH. In particular, there was a similar prevalence of diastolic dysfunction (15.5% vs 16%; *P* = .93) and similar left atrial diameter size (3.7 ± 0.7 vs 3.6 ± 0.7 cm; *P* = .29). The prevalence of RV dysfunction was similar between the 2 groups whether we defined this by any RV dysfunction (78.2% vs 70.3%; *P* = 0.25) or separated into mild/moderate (55.2% vs 46.7%; *P* = .36) and severe (19.0% vs 17.6%; *P* = 0.90) (Table 1).

RHC data were compared between drift and nondrift cohorts at diagnostic and follow-up RHC (Table 2). At diagnostic RHC, both groups had similar resting baseline hemodynamic severity measured by cardiac index, PVR, right atrial pressure, and PCWP. Differences were noted between the drift and nondrift cohorts with provocative maneuvers; the drift cohort saw a statistically significant higher absolute change in PCWP during fluid challenge (5.1 ± 3.7 vs 2.9 ± 4.4 mm Hg; *P* < 0.01). However, there was no significant difference between groups in the presence of a positive fluid challenge (22.9% vs 13.7%; *P* = .19) (Table 2) that would have suggested differences in baseline presence of diastolic dysfunction of the left ventricle.

**Table 2:** Comparison of Diagnostic and Follow-Up RHC Data Between Drift and Nondrift Cohorts

	All PAH	Drift	Nondrift	*P* Value
	(N = 257)	(n = 58)	(n = 199)	
**RHC Hemodynamics**				
**Interval time, y**	4.1 [3.5]	4.9 [4.1]	3.8 [3.3]	.03
**Diagnostic**				
mRAP, mm Hg	8.5 [5.4]	9.4 [5.7]	8.2 [5.3]	.17
mPAP, mm Hg	50.6 [13.1]	50.9 [14.3]	50.6 [12.7]	.87
PCWP, mm Hg	9.0 [3.5]	9.5 [3.5]	8.8 [3.5]	.17
PFC, %	15.8	22.9	13.7	.19
ΔPCWP with FC, mm Hg	2.8 [4.2]	5.1 [3.7]	2.9 [4.4]	< .01
PVR, WU	10.0 [5.3]	9.4 [5.0]	10.2 [5.4]	.36
CI, Fick	2.4 [0.9]	2.4 [1.2]	2.4 [0.8]	.58
CO, Fick	4.4 [1.6]	4.7 [2.1]	4.3 [1.4]	.11
**Follow-up**				
mRAP, mm Hg	9.1 [5.5]	13.1 [5.3]	7.9 [5.0]	< .01
mPAP, mm Hg	47.6 [13.0]	50.5 [15.5]	46.8 [12.1]	.05
PCWP, mm Hg	11.4 [5.7]	19.4 [3.9]	9.0 [3.7]	< .01
ΔPCWP from index RHC, mm Hg	2.4 [6.4]	9.9 [5.5]	0.3 [4.8]	< .01
PVR, WU	8.6 [4.8]	7.9 [5.4]	8.8 [4.6]	.27
CI, Fick	2.5 [0.7	2.3 [0.7]	2.5 [0.7]	.05
CO, Fick	4.5 [1.5]	4.4 [1.3]	4.6 [1.5]	.49

Data are presented as mean [SD] or as otherwise indicated. CI = cardiac index; CO = cardiac output; Interval time = median time period between diagnostic and follow-up RHC; mRAP = mean right atrial pressure; mPAP = mean pulmonary arterial pressure; PAH = pulmonary arterial hypertension; PCWP = pulmonary capillary wedge pressure; ΔPCWP = change in wedge pressure between index and follow-up RHC; ΔPCWP with FC = change in wedge pressure with fluid challange. PFC = positive fluid challenge; PVR = pulmonary vascular resistance; RHC = right heart catheterization; WU = Wood units.

**Figure 2:**
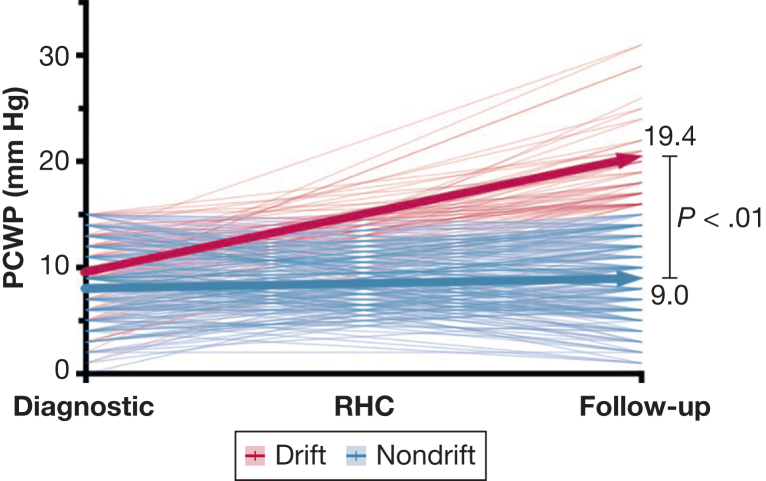
Individual and average change in PCWP between diagnostic and follow-up RHC between drift and nondrift populations. PCWP = pulmonary capillary wedge pressure; RHC = right heart catheterization.

On follow-up RHC, the drift cohort had a significantly higher resting baseline PCWP (19.4 ± 3.9 vs 9.0 ± 3.7 mm Hg; *P* < .01) and absolute change in PCWP when comparing with resting baseline at diagnosis (9.9 ± 5.5 vs 0.3 ± 4.8 mm Hg; *P* < .01) (Fig 2, Table 2). The drift cohort also had a significantly higher mean right atrial pressure (13.1 ± 5.3 vs 7.9 ± 5.0 mm Hg; *P* < .01) on follow-up RHC.

The time from diagnostic to follow-up RHC differed by an average of 1 year in the drift and nondrift cohorts (4.9 ± 4.1 vs 3.8 ± 3.3 years; *P* = .03). Therefore, we sought to ensure that observed differences in follow-up data were not due to an extended time to develop comorbidities tightly associated with aging. After adjusting for follow-up time, the hemodynamic measurements of right atrial pressure, PAP, PCWP, and delta pulmonary capillary wedge pressure (ΔPCWP) remained significantly different between groups (e-Table 1).

Use of PAH-specific therapy was nearly universal and did not differ between the drift and nondrift cohorts (100% vs 98.5%; *P* = .35). The percent of patients on phosphodiesterase type 5 inhibitors, endothelin receptor antagonists, and soluble guanylate cyclase stimulator therapy for drift was 87.9%, 72.4%, and 1.7%, respectively. The corresponding percentage for the nondrift group was 88.9%, 76.9%, and 3.0%, respectively (Table 3). These frequencies represent use over the entire study period and do not necessarily indicate what a given patient was receiving at a single time point. Of note, use of prostacyclins was common and similar in both the drift and nondrift cohorts (82.8% vs 80.4%, respectively). Not all patients were on parenteral therapy. Additionally, reflecting the extended period of time patients were followed, many were placed on multiple forms of prostacyclin throughout the study period (eg, oral, inhaled, subcutaneous, parenteral). Patients on multiple forms of prostacyclin therapy are included in the mixed category. Reflecting changing therapeutic guidelines throughout the study period, most patients were initiated on monotherapy as opposed to combination therapy in both drift (79% vs 21%) and nondrift (84% vs 16%) (e-Table 2). Of note, use of diuretics was common and not significantly different between drift and nondrift cohorts (100% vs 94.5%; *P* = .06) (Table 3).

**Table 3:** Comparison of Pulmonary Hypertension Specific Pharmacologic Therapy and Outcomes Between Drift and Nondrift Cohort

	All PAH	Drift	Nondrift	*P* Value
	(N = 257)	(n = 58)	(n = 199)	
**Therapy**				
Any PAH therapy	254 (98.8)	58 (100)	196 (98.5)	.35
Any prostacyclin	208 (80.9)	48 (82.8)	160 (80.4)	.69
Oral	7 (3.4)	2 (4.2)	5 (3.1)	NA
Inhaled	9 (4.3)	2 (4.2)	7 (4.4)	NA
Subcutaneous	2 (1.0)	0 (0)	2 (1.2)	NA
IV	60 (28.8)	13 (27.1)	47 (29.4)	NA
Mixed	130 (62.5)	31 (64.5)	99 (61.9)	NA
ERA	195 (75.9)	42 (72.4)	153 (76.9)	.49
PDE-5	228 (88.7)	51 (87.9)	177 (88.9)	.83
Soluble guanylate cyclase stimulator	7 (2.7)	1 (1.7)	6 (3.0)	.59
Diuretic use^a^	246 (95.7)	58 (100)	188 (94.5)	.06
**Outcomes**				
Time-to-first hosp., d				
Any cause	599 (151-1,355)	555 (165-1,941)	615 (151-1,286)	.22
PAH-specific	718 (194-1,734)	708 (295-2,028)	765 (178-1,625)	.65

Data are presented as median (interquartile range), No. (%), or as otherwise indicated. ERA = endothelin receptor antagonist; hosp. = hospitalization; Mixed = patients on multiple forms of prostacyclin therapy throughout the study period; PAH = pulmonary arterial hypertension; PDE-5 = phosphodiesterase-5 inhibitor.

a Diuretic use includes the following medications: bumetanide, furosemide, torsemide, metolazone, spironolactone, hydrochlorothiazide, eplerenone, and amiloride.

**Figure 3:**
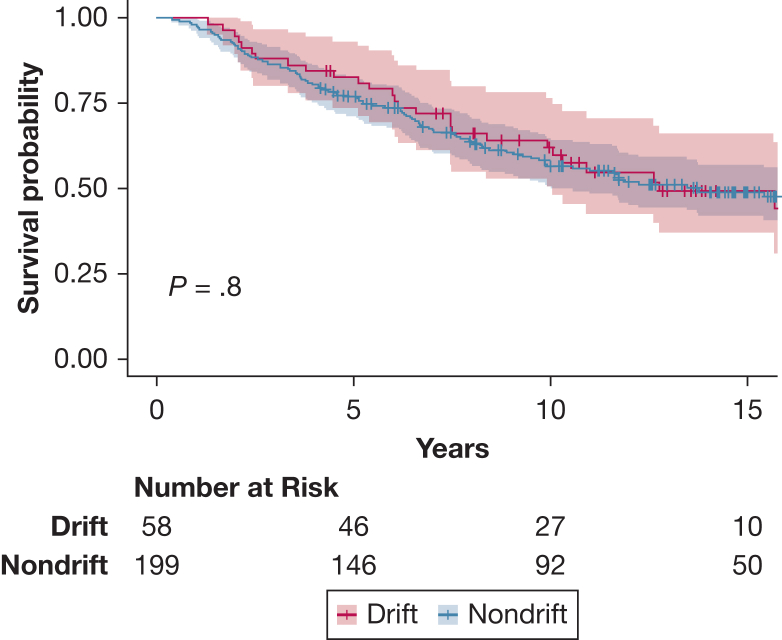
Kaplan-Meier curve for comparison of overall survival between drift and nondrift populations. Overall survival was defined as time from diagnostic RHC to death from any cause. RHC = right-heart catheterization.

There was no significant difference in unadjusted overall survival between the drift and nondrift cohorts (log-rank test, *P* = .80) (Fig 3). The time to first hospitalization after initiation of PAH therapy for any cause (555 vs 615 days; *P* = 0.22) and for clinical decompensation specifically due to PH (708 vs 765 days; *P* = .65) was also the same between the drift and nondrift groups (Table 3). We performed Cox proportional regression analysis adjusting for the covariates age, sex, race, baseline PCWP, AF, BMI, and diabetes mellitus type 2. This analysis did not change statistical significance for survival or hospitalization outcomes (e-Table 4). Because guidelines in care and management changed over our time period of inclusion, we sought to determine if there were any period affects that would change the significance of our results. We thus stratified patients into those who were diagnosed before 2007 and those who were diagnosed in 2007 or after. Stratification based on timing of diagnostic RHC did not change the significance of our results (log-rank test < 2007, *P* = .82; log-rank test ≥ 2007, *P* = .58) (e-Fig 1).

## Discussion

In this longitudinal, retrospective cohort study, we found that nearly one-fourth of patients with PAH develop elevation in their PCWP over a median follow-up of 4.1 years. Patients who experienced phenotypic drift were more likely to be Black, have AF, and have comorbidities associated with metabolic syndrome including increased BMI and insulin resistance. However, the 2 cohorts were not distinguishable by resting baseline hemodynamics and echocardiographic features. Overall survival and time to hospitalization did not differ significantly between the groups, suggesting that the development of hemodynamic features of group 2 PH did not impact outcomes in this cohort.

There has been recent interest in the concept of phenotypic drift. In their retrospective cohort study, Harder et al^19^ used group-based trajectory modeling to separate patients with PAH into those who develop high PCWP and those who maintained low PCWP over the course of 10 years. They found a similar proportion of enrollees, approximately one-fourth, followed the high PCWP trajectory and developed a group 2 PH phenotype over time. Furthermore, they also identified enrichment for diabetes, increased BMI, and AF in the high PCWP cluster. However, they identified an initial PCWP ≥ 12 mm Hg and presence of left atrial enlargement as predictors of a high trajectory, whereas routine hemodynamics and imaging, including these variables, did not differ between our study’s drift and nondrift groups at baseline. Our results build on the literature of understanding phenotypic drift in different hemodynamic subtypes and highlight the difficulty of using baseline characteristics to identify patients at risk for phenotypic changes.^20^ They also reported worse unadjusted survival in their high PCWP cohort, whereas we saw no difference in survival in our study’s phenotypic drift population. A key difference between this approach and our own was our use of a standard, clinically used PAH definition throughout the study period, with a PCWP ≤ 15 mm Hg defining PAH, as opposed to their focus of clusters above and below PCWP of 12 mm Hg. Thus, our data identify the risk of developing phenotypic drift based on standard clinical characteristics and show that imaging and resting baseline hemodynamics are not different in a group of severe PAH. Furthermore, they had lower overall rates of pulmonary vasodilator usage, including lower rates of combination therapy. Our study thus facilitates novel observations about phenotypic drift in the context of aggressive PAH therapies. Differences in survival in the Harder et al^19^ paper may be related to differences in treatment patterns and even perceived risk of group 2 PH, which was not present in this cohort because the therapies used were similar between the drift and nondrift populations.

The 2022 European guidelines^21^ suggest initial monotherapy for patients who have PAH and CV risk factors. Evidence for this is limited and occasionally conflicting. A cluster analysis from the Comparative, Prospective Registry of Newly Initiated Therapies for Pulmonary Hypertension (COMPERA) suggests that although patients with PAH both with and without CV risk factors derive treatment response, those without CV comorbidities had a more robust response to therapy in terms of improvement in 6-minute walk distance, World Health Organization functional class, serum bone natriuretic peptide, and survival.^10^ However, treatment strategies between clusters with and without CV comorbidities differed, and those with more traditional PAH phenotypes may have been treated more aggressively, accounting for the observed differences. More recent research corroborates this practice, suggesting patients with PAH and CV risk factors are in fact less likely to receive initial combination therapy.^4^^,^^19^^,^^22^ Additionally, an analysis of published studies in PAH suggests that patients with 1 to 2 CV comorbidities enjoy benefits of combination therapy in PAH that are similar to counterparts without CV comorbidities.^23^ Our data argue that patients with CV comorbidities at diagnosis and those that go on to experience phenotypic drift into CpcPH have similar outcomes compared with their cohorts without these features. This conclusion includes patients receiving prostacyclin therapy, suggesting there may be a role for this aggressive therapy in patients with a new diagnosis of PAH regardless of the long-term risk for group 2 PH.

It remains unclear whether patients who experience phenotypic drift have occult group 2 PH at diagnosis or whether they develop features of left heart disease over time. Because we defined cohorts using resting baseline PCWP, we cannot be sure that euvolemic individuals with group 2 PH and normal resting baseline PCWP were not misclassified. However, all patients in this cohort had marked elevation in PVR and nearly all received aggressive PAH therapy, including parenteral prostacyclins in many cases. This observation lends confidence that this cohort was a precapillary PAH cohort at baseline. Furthermore, rates of PAH therapies in the 2 groups did not differ, suggesting that increases in PCWP were likely not caused by PAH therapy itself eliciting pulmonary vascular congestion in a population with prior group 2 PH. Although an exploratory outcome only, the significant difference between cohorts in absolute rise of PCWP with fluid challenge suggests that use of provocative maneuvers at diagnosis can help distinguish people at risk for developing group 2 PH features, even in the setting of traditional thresholds for positive fluid challenge not being different. These demographic and hemodynamic differences at diagnosis suggest a distinct pathophysiology at onset in the phenotypic drift population. Whether this pathophysiology is related to pulmonary vascular disease or comorbidities is not determined.

Our study had several strengths. This was a large real-world cohort with long-term follow-up > 10 years in many cases. Treatment strategies thus reflect modern practice patterns, and with high use of prostacyclin therapy, included patients with a high confidence for having PAH. Several studies have analyzed outcomes between patients with PAH with and without CV comorbidities, but few have looked directly at changes in PCWP over time and how this affects outcomes as we did.

A number of limitations to our data exist. We had incomplete data regarding a number of potentially important variables including the following: (1) functional outcomes (eg, 6-minute walk distance) to compare groups using standard clinical risk scores, (2) presence of obstructive and restrictive lung diseases due to missing pulmonary function testing data, and (3) RHC tracings and echocardiographic images for manual review, which is an important component of real-world practice to ensure accuracy of hemodynamics and echocardiographic reports. This is an especially important consideration when interpreting PCWP during RHC, as standard of practice regarding measuring this value remains under debate and has changed over the course of this study. However, all patients in this cohort were treated by expert PAH clinicians who independently review RHC tracings and echocardiographic images to incorporate into treatment decisions. Another limitation is that we do not know the indication for those patients with PAH undergoing follow-up RHC and are thus not able to determine whether this was done for clinical worsening or during routine care. Because of this, there may be referral bias for those in this study cohort who underwent follow-up RHC. Although patient populations as related to demographics, comorbidities, RHC hemodynamics, and transthoracic echocardiogram findings did not significantly differ on most accounts between those who had a follow-up RHC and those who did not (e-Table 3), residual selection bias may still remain. This cohort was from a single academic institution in the United States, which may limit the generalizability of results. However, patient characteristics in this institutional cohort are similar to that described in modern US registries and similar to those published from other single-center cohorts, suggesting our data may be applied more broadly.^3^^,^^4^^,^^24^ Although we think the prolonged nature of follow-up is an overall strength of this study, there were also multiple changes in guidelines and care during this time period. Our stratified analysis attempts to address these concerns; however, residual bias as it relates to changes in care must be considered when interpreting our data. Additionally, our time to hospitalization analysis assumes patients were admitted within our health system and does not account for hospitalizations elsewhere. Specifics on timing of initiation or discontinuation of therapies including parenteral prostacyclins are not available. Knowing rates of discontinuation of IV prostacyclin use, or need for escalation from oral to IV therapy, for example, would provide additional insightful information on clinical improvement and worsening between groups. Finally, the retrospective nature of the study also brings forth unmeasured confounding and selection bias not otherwise accounted for. Prospective work in the area will need to be done to support our results.

## Interpretation

Phenotypic drift defined by newly elevated PCWP is common in patients with initial diagnosis of PAH. Although associated with AF and features of metabolic syndrome, baseline hemodynamics and imaging characteristics cannot be used to identify those at risk for phenotypic drift. Those who experience this phenomenon have similar outcomes to those who do not when treated similarly. Our findings suggest that the risk for phenotypic drift should not routinely inform initial treatment decisions in PAH.

## Funding/Support

This study was supported by R01 10.13039/100023110HL142720, K24 HL155891, R01 HL 155278, R01 FD 007627, R01 HL 163960, R01 HL146588.

## Financial/Nonfinancial Disclosures

A. R. H. has served as consultant to Gossamer Bio, United Therapeutics, Johnson & Johnson, Merck and Tenax Therapeutics. She is a stockholder at Tenax Therapeutics and an editorial board member with *CHEST*.

None declared (K. T. S., J. A., J. D. G., H. N., E. L. B.).

## Supplementary Data

e-Online Data
