# Supplementary material for: Incidence and Clinical Associations With Phenotypic Drift From Pulmonary Arterial Hypertension to Combined Precapillary and Postcapillary Pulmonary Hypertension
Source: CHEST Pulm. 2025 Aug 19;3(4):100206. doi: 10.1016/j.chpulm.2025.100206 (PMC13419258; doi:10.1016/j.chpulm.2025.100206)
Supplement: e-Online Data [file mmc1.docx]

**Incidence and Clinical Associations with Phenotypic Drift from Pulmonary Arterial Hypertension to Combined Pre- and Post-capillary Pulmonary Hypertension**

**Kevin T. Schwalbach^1^(MD), Jeffrey Annis^2,3^(PhD), Jonah Garry^2^ (MD), Hui Nian^4^ (PhD), Evan Brittain^2^ (MD), Anna Hemnes^1^ (MD)**

**Supplemental Material**

**Supplemental Methods**

***PAH Algorithm:*** This is a machine learning algorithm that uses International Classification of Disease (ICD) 9/10 codes, Current Procedural Terminology (CPT) codes, and Food and Drug Administration (FDA) approved PAH medications to identify potential PAH patients^1^.

***PCWP during RHC:*** Standard practice at our institution for an individual patient is that wedge pressure is recorded according to a computer algorithm as the average between peak and trough of the “a wave.” This is done at end-expiration. It is a single measurement and not averaged over several cardiac cycles and is chosen by a digital algorithm. However, this can be overridden by our experienced proceduralist if there appears to be an error in the computer-selected waveform. Our PAH algorithm extracts these reported PCWP pressure from the EMR and these are the values used in analysis. We have previously published a comparison of electronic medical record reported PCWP vs interpretation by a pulmonary hypertension physician in 116 subjects and found excellent correlation^2^.

***Provocative RHC testing***: A “positive fluid challenge” was defined as an increase in PCWP to >18 mmHg^2^. Clinicians in our practice may choose to assign a primary diagnosis of PAH regardless of fluid challenge results if resting baseline hemodynamics supported this diagnosis and treat according to contemporary guidelines as such^3,4^.

***Comorbidity data:*** Machine learning algorithm that utilizes one or more of the following: *ICD‐9* and *ICD‐10* codes, CPT codes, medications, labs, or natural language processing algorithms on problem notes to identify coronary artery disease (CAD), atrial fibrillation (AF), diabetes mellitus (DM), obstructive sleep apnea (OSA), scleroderma, and hypertension (HTN). Lipid panels obtained from the synthetic derivative were not required to be fasting. We also used hemoglobin A1c >6.5% to define diabetes and between 5.7% - 6.4% to define pre-diabetes^1^.

***Echocardiographic Measurements***: The RV function was reported as normal, mild, moderate, severe. These measures are visual, semi-quantitative. The diastolic function are graded 1-4 based on the reader’s interpretation of the American Society of Echocardiography guidelines for grading diastolic dysfunction. Diastolic function was then dichotomized based on presence or absence of diastolic dysfunction regardless of severity in our data set. The LVEF was visual estimate. All values were determined by expert readers in clinical practice incorporating qualitative measures and visual recognition.

**Supplemental Tables**

**Sensitivity Analysis Correcting for Differences in Follow-up Time Between RHC**

**Effect 95% CI P-Value**

**mRAP** 4.78 [3.28 – 6.28] <0.01

**mPAP** 4.15 [0.61 – 7.7] 0.02

**PCWP** 10.23 [9.12 – 11.4] <0.01

**mPVR** 0.12 [-1.40 – 1.64] 0.87

**CI** -0.19 [-0.43 – 0.05] 0.12

**CO** -0.19 [0.66 – 0.27] 0.42

***e-Table 1*** – Results of sensitivity analysis correcting for time between diagnostic and follow-up RHC. Multivariable linear regression model was performed to include baseline measurements in addition to follow-up days as nonlinear terms. When correcting for follow-up time, the effect for RAP, PAP and PCWP remain significantly different between Drift and Non-Drift populations. mRAP = mean Right Atrial Pressure. mPAP = mean Pulmonary Artery Pressure. PCWP = Pulmonary Capillary Wedge Pressure. PVR = Pulmonary Vascular Resistance. CI = Cardiac Index by Fick. CO = Cardiac Output by Fick.

**Initial Treatment Type and Strategy**

**All PAH Drift Non-Drift**

(n = 257) (n = 58) (n = 199)

**Initial Monotherapy** 213 46 167

PDE5-I 94 19 75

ERA 39 8 31

Prostacyclin 80 19 61

**Initial Combination**  44 12 32

PDE5-i/ERA 8 2 6

Prostacyclin/ERA 20 7 13

Prostacyclin/PDE5-I 16 3 13

***e-Table 2*** *–* Frequency and type of initiation of monotherapy versus combination therapy between Drift and Non-Drift groups.

**All** **PAH** **PAH PAH P-value**

**w/ baseline RHC w/ follow-up RHC w/o follow-up RHC**

**(N = 415) (N = 257) (N = 158)**

**Age (y)** 51.2 [41.5-60.7] 50.6 [40.9-59.1] 52.7 [42.2-63.2] 0.07

**Female sex, N (%)** 310 (74.7) 198 (77.0) 112 (70.9) 0.20

**Race, N (%)**

White 324 (78.8) 204 (79.4) 120 (77.9) 0.77

Black 71 (17.3) 45 (17.5) 26 (16.9) 0.82

Other 16 (3.9) 8 (3.1) 8 (5.2) 0.26

**Co-Morbidities, N (%)**

AF 27 (6.5) 14 (5.4) 13 (8.2) 0.28

Scleroderma 86 (20.7) 46 (17.9) 40 (25.3) 0.08

OSA 44 (10.6) 25 (9.7) 19 (12) 0.41

Stroke 6 (1.4) 2 (0.8) 4 (2.5) 0.15

Prediabetes 77 (33.8) 52 (33.5) 25 (34.2) 0.91

Diabetes 51 (22.7) 34 (21.9) 17 (23.3) 0.82

CAD 62 14.9) 37 (14.4) 25 (15.8) 0.58

HTN 107 (25.8) 69 (26.8) 38 (24.1) 0.47

ESRD 28 (6.7) 14 (5.5) 14 (8.9) 0.11

Cirrhosis 39 (9.4) 24 (9.3) 15 (9.5) 0.99

**Lab Data**

BMI 27.9 [23.9-32.6] 28.5 [24.5-33.4] 27.2 [23.3-31.9] 0.04

BNP 274 [93.5-659.5] 203 [62.5-542.5] 326 [91-671] 0.99

Cr 0.96 [0.81-1.15] 0.96 [0.83-1.14] 0.95 [0.8-1.2] 0.34

eGFR 72.0 [58.0-88.5] 72.0 [60-87.2] 75.3 [55.7-89.5] 0.58

Glucose 93 [83-107] 92 [82-107] 92 [85-104] 0.38

A1c 5.8 [5.4-6.3] 5.8 [5.3-6.2] 5.7 [5.4-6.4]

HDL 42 [31-52] 42 [31-52] 45 [32-55] 0.79

LDL 94.5 [71-125] 93 [68.5-116.5] 98 [73.5-129.5] 0.71

Tgs 109.4 [83-159] 109 [84-160] 109.5 [79.5-163] 0.56

**TTE**

LVEF (%) 56.0 +6.3 55.5 +6.7 57 +5.3 0.02

LA Diameter (cm) 3.6 +0.7 3.6 +0.7 3.7 +0.7 0.57

RVSP (mmHg) 78.7 +24.5 79.3 +23.8 78.2 +26.5 0.44

RV dysfunction, N (%)

Any 262 (72.3) 171 (72.2) 93 (72.1) 0.98

Mild/Moderate 191 (52.3) 125 (48.6) 66 (51.2) 0.80

Severe 73 (20.1) 46 (17.9) 27 (20.1) 0.77

Diastolic

Dysfunction N (%) 75 (18.1) 41 (15.9) 34 (21.5) 0.12

**RHC Hemodynamics**

mRAP (mmHg) 8.9 +5.6 8.5 +5.4 9.5 +5.5 0.08

mPAP (mmHg) 49.8 +12.7 50.6 +13.1 48.9 +12.2 0.29

PCWP (mmHg) 8.9 +3.5 9.0 +3.5 9.1 +3.6 0.47

PVR (wu) 10.0 +5.2 10.0 +5.3 10.3 +5.3 0.44

CI (fick) 2.4 +0.9 2.4 +0.9 2.4 +0.9 0.98

CO (fick) 4.4 +1.8 4.4 +1.6 4.4 +1.9 0.84

***eTable 3 -*** Comparison of PAH patients who had follow-up RHC and those who did not have follow-up RHC.

AF = atrial fibrillation, OSA = obstructive sleep apnea, CAD = coronary artery disease, HTN = hypertension, CKD = Chronic Kidney Disease, ESRD = End Stage Renal Disease, BMI = body mass index, BNP = B-type natriuretic peptide, Cr = creatinine, eGFR = estimated glomerular filtration rate, HDL = high-density lipoprotein, LDL = low-density lipoprotein, Tgs = Triglycerides, LVEF = left ventricular ejection fraction, LA = left atrial, RVSP = right ventricular systolic pressure, RV = right ventricle. mRAP = mean right atrial pressure, mPAP = mean pulmonary arterial pressure, PCWP = pulmonary capillary wedge pressure, PVR = pulmonary vascular resistance, CI = cardiac index, CO = cardiac output. Data are presented as mean +standard deviation, median [interquartile range], or number (percent).

**Cox-Proportional Regression Analysis for Time-To-Hospitalization**

**Coefficient SE Wald Z P-value**

**Group (non-drift)** -0.06 0.18 0.34 0.74

**Age** -0.01 0.01 -0.03 0.98

**Age (nonlinear)** -0.01 0.02 -0.43 0.67

**Sex (Female)** 0.12 0.18 0.62 0.54

**Race (White)** -0.05 0.18 -0.29 0.77

**PCWP** 0.06 0.05 1.25 0.21

**PCWP (nonlinear)** -0.11 0.06 -1.66 0.09

**A. Fib**  0.38 0.38 1.00 0.32

**DM** 0.60 0.24 2.51 0.01

**BMI** -0.02 0.03 -0.83 0.41

**BMI (nonlinear)** 0.02 0.03 0.50 0.62

***4a.***

**Cox-Proportional Regression Analysis for Survival**

**Coefficient SE Wald Z P-value**

**Group (non-drift)** -0.03 0.22 **-**0.12 0.9

**Age** 0.05 0.02 2.53 0.01

**Age (nonlinear)** -0.04 0.02 -1.84 0.07

**Sex (Female)** 0.11 0.22 0.50 0.62

**Race (White)** 0.48 0.26 1.83 0.07

**PCWP** -0.04 0.06 -0.76 0.45

**PCWP (nonlinear)** 0.04 0.07 0.54 0.59

**A. Fib** 0.21 0.42 0.52 0.61

**DM** 0.51 0.27 1.88 0.06

**BMI** -0.04 0.04 -1.18 0.24

**BMI (nonlinear)** 0.05 0.04 1.10 0.27

***4b.***

***eTable 4*** – Results of adjusted analysis using cox-proportional regression correcting for age, sex, race, mPCWP, AF, DM, and BMI in both time-to hospitalization due to PH specific decompensation (a) and overall survival (b). Adjustment did not change statistical significance of our outcomes. PCWP = Pulmonary Capillary Wedge Pressure. A. Fib = atrial fibrillation. DM = diabetes mellitus. BMI = body mass index.

**Hospitalization and Survival in Patients Diagnosed Before 2007**


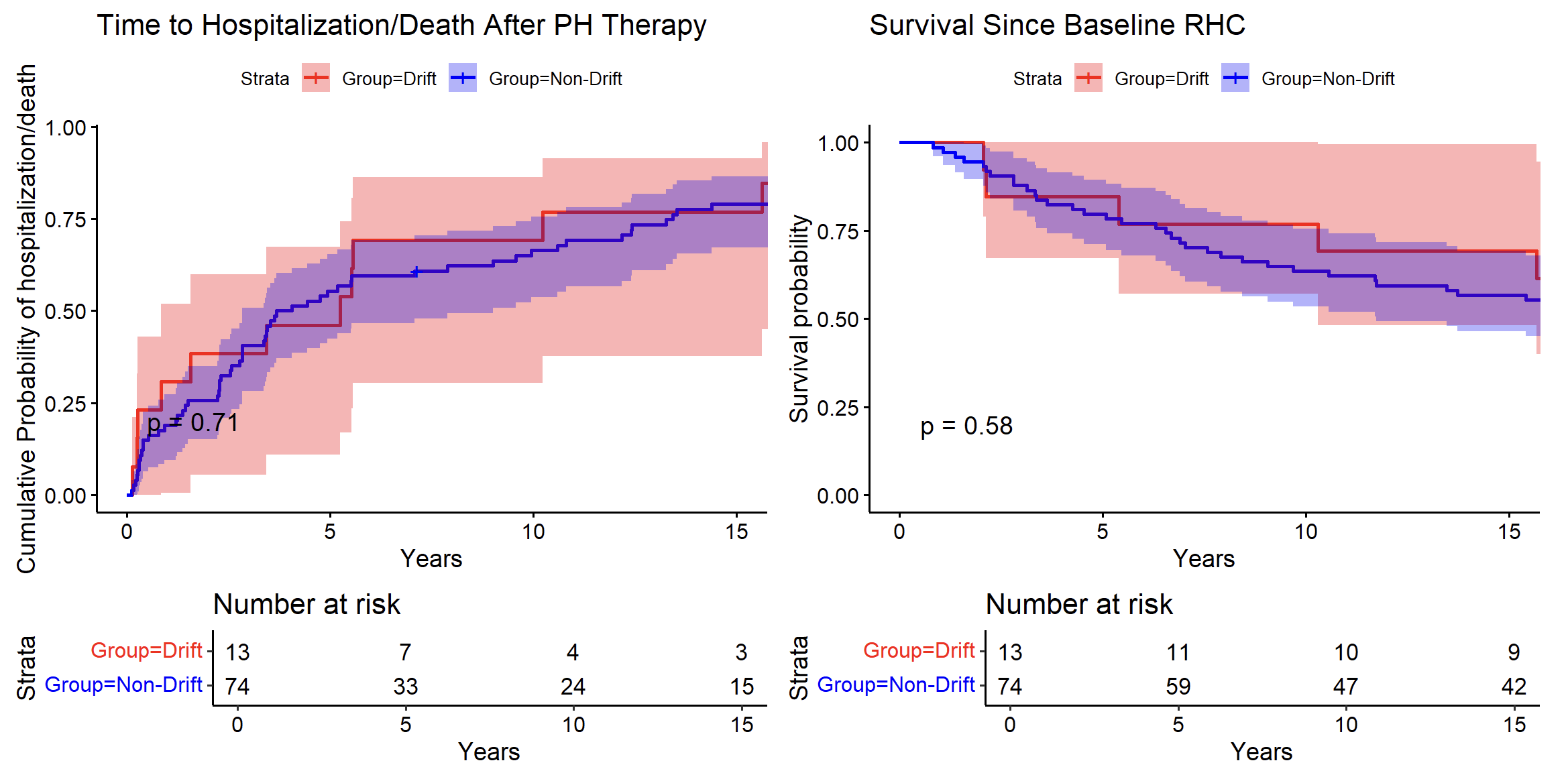


***1a***

**Hospitalization and Survival in Patients Diagnosed in or After 2007**


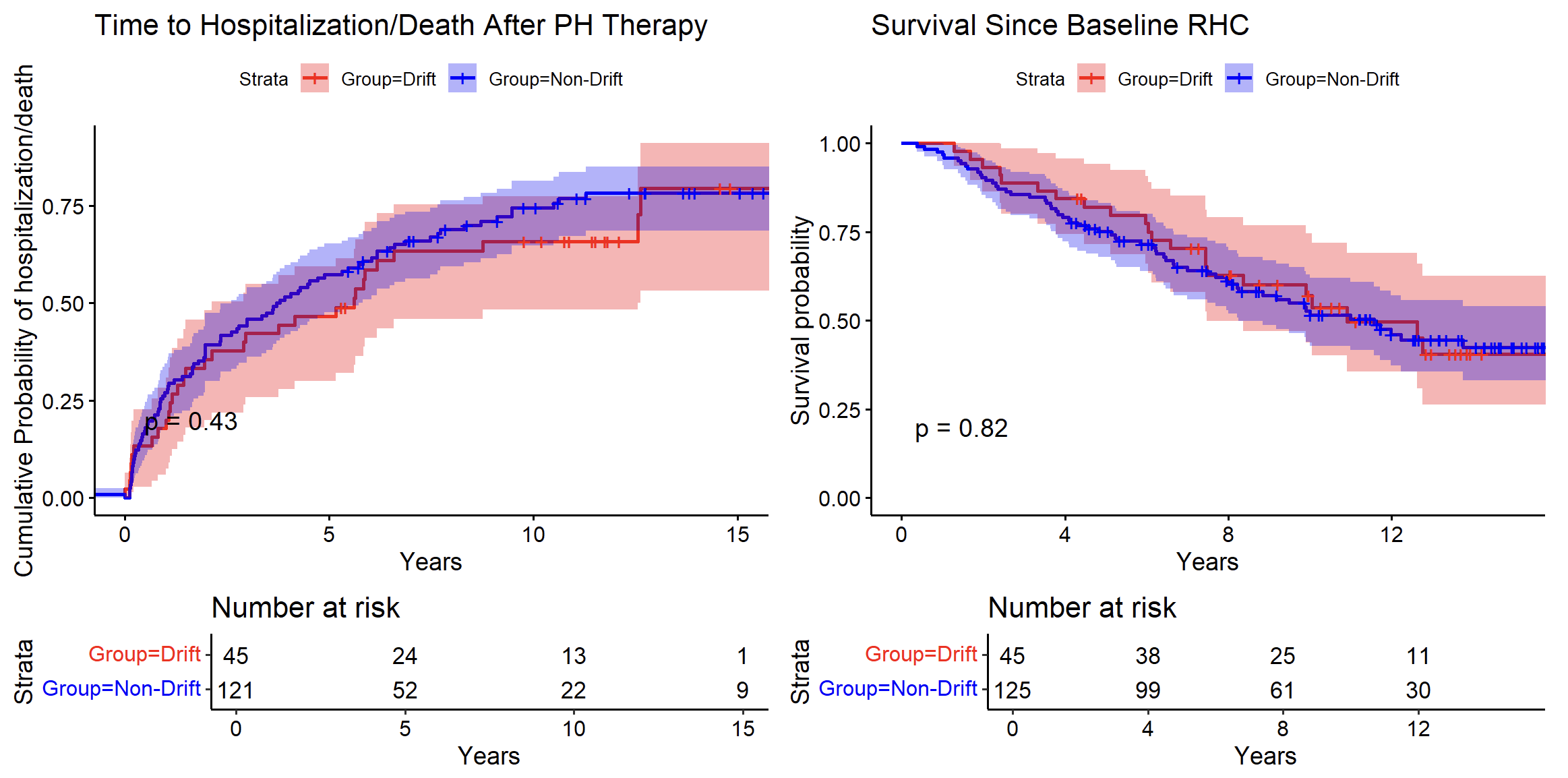


***1b***

***eFigure 1****. Period analysis.* Kaplan-Meier Curves comparing Time-to-Hospitalization and Overall Survival between Drift and Non-Drift Populations stratified by diagnostic RHC. Patients diagnosed before 2007 are shown in *eFigure 1a*. Those diagnosed in the year 2007 or after are shown in *eFigure 1b*. Stratifying by date of diagnosis did not change statistical significance.

***Supplemental References***

(1) Schuler KP, Hemnes AR, Annis J, et al. An algorithm to identify cases of pulmonary arterial hypertension from the electronic medical record. *Respir Res*. 2022 May 28;23(1):138.

(2) Assad TR, Brittain EL, Wells QS, Farber-Eger EH, Halliday SJ, Doss LN, Xu M, Wang L, Harrell FE, Yu C, Robbins IM, Newman JH, Hemnes AR. Hemodynamic evidence of vascular remodeling in combined post- and precapillary pulmonary hypertension. Pulm Circ. 2016 Sep;6(3):313-21.

(3) Fujimoto N, Borlaug BA, Lewis GD, et al. Hemodynamic responses to rapid saline loading: the impact of age, sex, and heart failure. *Circulation*. 2013 Jan 1;127(1):55-62.

(4) Robbins IM, Hemnes AR, Pugh ME, et al. High prevalence of occult pulmonary venous hypertension revealed by fluid challenge in pulmonary hypertension. *Circ Heart Fail*. 2014 Jan;7(1):116-22.
